# A faecal exposure assessment of farm workers in Accra, Ghana: a cross sectional study

**DOI:** 10.1186/s12889-016-3266-8

**Published:** 2016-07-16

**Authors:** Prince Antwi-Agyei, Adam Biran, Anne Peasey, Jane Bruce, Jeroen Ensink

**Affiliations:** Department of Disease Control, Faculty of Infectious and Tropical Diseases, Environmental Health Group, London School of Hygiene and Tropical Medicine, Keppel street, London, WC1E 7HT UK; Department of Epidemiology and Public Health, University College London, 1-19 Torrington Place, London, UK; Department of Disease Control, Faculty of Infectious and Tropical Diseases, London School of Hygiene and Tropical Medicine, Keppel street, London, UK

**Keywords:** Wastewater use, Faecal exposure, Urban agriculture, Farmers, Ghana

## Abstract

**Background:**

Wastewater use in urban agriculture is common as a result of rapid urbanisation, and increasing competition for good quality water. In order to minimize risks to farmers and consumers of wastewater irrigated produce the World Health Organization (WHO) has developed guidelines for the safe use of wastewater in agriculture. These guidelines are based on a Quantitative Microbial Risk Assessment (QMRA) model, though the reliability of this model has been questioned due to a lack of primary data. This study aimed to assess the ability of the WHO guidelines to protect farmers’ health, by identifying and quantifying key exposures associated with the transmission of faecal pathogens in wastewater irrigated agriculture.

**Methods:**

Eighty farmers were observed and interviewed during the dry and wet seasons, and water and soil samples were analysed for the presence of *E. coli*. STATA 12 was used for descriptive analyses of farmers’ exposure and risk practices, and also to determine risk factors for soil and irrigation water contamination, while the WHO QMRA model and @Risk 6 were used to model farmers’ infection risk to pathogens.

**Results:**

The results showed that although irrigation water was highly contaminated (5.6 Log *E. coli*/100 ml), exposure to farm soil (2.3 Log *E. coli*/g) was found to be the key risk pathway due to soil-to-mouth events. During the observations 93 % of farmers worked barefoot, 86 % experienced hand-to-soil contact, while 53 % experienced ‘soil’-to-mouth events, while no ‘water’ to mouth contacts were observed. On average, farmers were found to have 10 hand-to-mouth events per day. From the indicator based QMRA model the estimated norovirus infection risk to farmers was found to be higher than guidelines set by the WHO.

**Conclusions:**

This study found exposure to soil as the critical pathway of pathogen risk in wastewater farmers, and that this risk exceeded recommended health targets. The study recommends the incorporation of hand-to-mouth events, the use of actual pathogen concentrations, and the use of direct exposure frequencies in order to improve the reliability of risk estimates from QMRA models.

## Background

The use of untreated, or partially treated wastewater in agriculture is common in countries with rapid and uncontrolled urban growth [1]. The exact extent of wastewater use in agriculture is unknown, but estimates range from between 4 to 24 million ha of agricultural land receiving wastewater [2, 3]. The use of wastewater for irrigation has been associated with health risks in farmers, and consumers of wastewater irrigated produce [4]. In order to minimize these health risks, the World Health Organisation (WHO) has developed guidelines for the safe use of wastewater in agriculture. These guidelines have been the focus of discussion for years and have seen several revisions with the most recent guidelines published in 2006 [1].

The current guidelines are based on a Quantitative Microbial Risk Assessment (QMRA) and use a similar approach to the WHO drinking water guidelines [5]. QMRA is used to estimate disease risks that should not exceed the maximum permissible disease burden of 10^-6^ disability-adjusted life years (DALYs) per person per year (pppy) arising from exposure to wastewater [1]. The risk estimate from QMRA is then used to determine the total pathogen reductions required to achieve the tolerable risk of infection due to a particular pathogen. Although useful, the reliability of estimates from QMRA depends on the quality of the input data describing the occurrence, persistence, human dose–response of pathogens in the environment, the mass or quantity of environmental media (e.g. soil and wastewater) exposed to humans, and on the time exposed to sources of hazards.

The concern with the wastewater guidelines QMRA is that they are based on many assumptions from different datasets [6]. For example, correlations of *E. coli,* or faecal coliforms to pathogens are used instead of actual concentrations of pathogens [7]. In addition most QMRA studies on occupational risk to farmers rely on approximations for the frequency, duration, and type of contact by farmers irrigating with wastewater, as well as the mass or volume of irrigation water or soil ingested per contact episode There is also a lack of data on the mass of soil ingested per contact event or for the different types of farming activities such as transplanting and weeding. In the QMRA model the number of days a farmer works in the field over a year equates to the number of days they are likely to accidentally ingest contaminated soil [6], though there is lack of evidence to support this. In addition the QMRA model assumes only a faecal-oral transmission route for pathogens in wastewater contaminated soil, not for exposure to water, nor for direct contact with contaminated soil, even though other modes of transmission are well established [8, 9]. The paucity of data for risk assessment therefore calls for more field-based data that can help validate and improve the accuracy and reliability of risk estimates from QMRA models. This paper presents the results of an exposure assessment that observed farmers’ exposure to wastewater, and wastewater irrigated soil in Accra, Ghana as part of their day-to-day farming activities. The study aimed to determine key exposures associated with the risk of transmission of faecal pathogens in farmers using wastewater for irrigation.

## Methods

In the period from October to December 2012 (dry season), and from June to August 2013 (wet season) farmers irrigating with wastewater in Accra, Ghana were observed and interviewed to identify risk behaviours and to quantify their contact time to faecal pathogens. In addition water and soil samples were collected and analysed for *E. coli* and human norovirus (results of virus data not presented in this paper).

### Study area

Accra is the capital city of Ghana with an estimated population of 1.9 million [10]. It is estimated that less than 6 % of Accra is connected to a sewerage system with the majority of the city reliant on onsite forms of sanitation like pit latrines and septic tanks [11]. There are over twenty wastewater treatment facilities in Accra, but only seven were reported to be functioning adequately as of 2010 [11]. There are seven major sites where wastewater in agriculture is used, with a total area of 160 ha [12]. Farmers at these sites apply water through watering cans using irrigation water sources that include drain water, channelled rivers and dugouts. The dugouts are man-made ponds used to store the various sources of water used for irrigation. The most commonly cultivated crops are salad vegetables including lettuce, cabbage, and spring onions. Three of the sites (Korle Bu, 12.3 ha, Dzorwulu, 8.2 ha, and Marine Drive, 3.5 ha) were selected for this study because of their size, the cultivation of salad crops normally consumed uncooked and the use of wastewater for irrigation.

### Data collection

#### Sample collection and analyses

Irrigation water and soil samples were collected between 7 am and 10 am when irrigation and key soil related farming activities were undertaken by farmers. Irrigation water samples were collected directly from open drains and dug-outs into sterile 500 ml Whirl-Pak bags using a sterile bailer. Samples were collected from where each of the 80 farmers was observed collecting water for irrigation. For all samples the site conditions including exposure to visible human faeces and proximity to garbage and latrines were recorded. At the laboratory serial dilutions of the raw sample with phosphate buffered saline (PBS) were prepared in sterile micro centrifuge tubes.

Farm soil samples were collected using a sterile metal spatula to a depth of 5 cm into 250 ml Whirl-pak bags until the bag was at least half full. A total of 7 soil samples were collected within an area of 3 m^2^ and combined into a single sample to increase sample representativeness [13]. All samples were placed in an ice-box and transported to the laboratory within 6 h of collection for processing. Samples were processed immediately or stored in a 4 °C refrigerator until ready for processing within 24 h.

At the laboratory soil samples were homogenised and 10 g of each sample was measured to which 20 ml of sterile PBS (pH, 7.2) was then added. The sample was vortexed for 30 s and shaken vigorously on a shaker for 30 min at room temperature. The sample was then allowed to settle for 15 min and 10 ml of supernatant transferred into a new sterile 50 ml tube for the *E. coli* assays. All laboratory staff were blinded on the sources of irrigation water, and farm soil in order to eliminate potential biases during sample analyses. Soil and water samples were analysed using the membrane filtration technique with BBL MI agar (Beckton Dickinson, Sparks, USA) to determine the prevalence and concentrations of *E. coli* [14]. Serial dilution ranges were pre-optimized to ensure that ranges allowed enumeration of roughly 95 % of samples per sample type. The method and results for the virus data have not been presented in this paper but can be found elsewhere [15].

The number of soil and irrigation water samples to be collected was calculated with STATA 12 (StataCorp LP, College Station, USA) and corresponded to the estimated number of farm produce samples to be collected at the farm as part of a related study [15].

#### Observations

At the farm sites 80 farmers who had at least one bed of ready-to-harvest lettuce had been recruited as part of a related farm-to-fork study where produce quality was assessed [15]. Farmers were randomly selected using their farm plots/beds as identification. Each farmer was observed continuously for three hours (7.00 am – 10.00 am) from October to December 2012. This study used a direct observation approach where researchers observed and recorded pre-specified behaviours as and when they occurred [16]. To reduce any form of bias, observers tried to be unobtrusive during the observation to minimise the effect of their presence on subjects’ behaviours. In addition, farmers were told that the observations were designed to learn more about their farming activities including irrigation and manure application (Table 1). Farmers were not told specifically that risk behaviours associated with faecal exposure were being documented.

**Table 1:** Operational definition of farm activities

Farm activity	Operational definition
Bed preparation	The use of hoe, rake and other farm implements to prepare a plot of ground or the soil (farm bed) for planting seedlings of salad crops.
Transplanting	The removal of seedlings from the nursery to be planted on the newly prepared beds
Weeding	The use of hands or hand-held knives to remove small weeds that have mixed with the salad crops
“Forking” (soil tilling)	The use of hand-held knife/fork to turn over the soil to allow air flow. This activity is often done alongside weeding.
Irrigation	The use of watering cans or water hose to apply water to the salad crops.
Manure application	Application of chicken manure with or without the use of protective clothing such as hand gloves

A structured observation guide was used to record behaviours while tally sheets were used to capture hand-to-mouth/face contact events. Exposure to faecal contamination was defined as direct contact with soil, irrigation water or both by hands, feet, mouth or face of the farmer without any protective clothing. Exposure to wastewater was defined as having direct contact with irrigation water. The total time farmers spent on each farm activity was recorded by indicating the start and end times. Similarly, the total time farmers worked unprotected and came into contact with either soil or irrigation water was recorded. In as much as possible, observation of farmers’ behaviour covered all the various stages of cultivation including bed preparation, transplanting, weeding, irrigating and harvesting. Farm workers access to water and sanitation were also observed as well as their food hygiene practices including hand washing. It must be noted that in as much as the observations were conducted only once and for only 3 h per farmer and hence might not reflect the actual exposure patterns, the aim of the study was to provide vital information on high risk farming activities, and the frequencies that farmers are actually in direct contact with faecal pathogens through wastewater or wastewater contaminated soil.

#### Questionnaire

Following the field observation, a questionnaire was verbally administered to gather background information including socio-economic and personal characteristics, the time and days spent undertaking different farm activities during the rest of year and the use and application of fertilizer. Information was also collected on availability of water supply (drinking and hand washing water), sanitation and hygiene practices at the farm.

#### Quantitative microbial risk assessment

The Microsoft Excel QMRA model developed by Mara and Sleigh [7] for the WHO guidelines was used to estimate the pathogen infection risk using the observed and reported exposure frequencies of farmers in this study (see Table 7 for more details). In the WHO model an exposure of 300 days per year is used (though flexible) for labour intensive agriculture; representing farming practices in low and middle-income countries. The model further assumes that between 10 and 100 mg of soil is accidentally ingested by farmers per day during their fieldwork. The model uses the Karavarsamis-Hamilton method [17] together with the norovirus dose–response model by Teunis et al. [18] and the 10,000 Monte Carlo simulations to estimate the norovirus infection risk among wastewater farmers for restricted irrigation. A maximum tolerable additional disease burden of 10^-6^ DALY per person per year (pppy) as used in the WHO guidelines was adopted in this study, which equates to a maximum permissible infection risk of 1.4 × 10^-3^ pppy for norovirus which is considered to pose the highest risk compared to bacteria and protozoans. The estimated risk in this study was compared to a relaxed DALY burden of 10^-4^ pppy as recommended by Mara et al. [19] which also equates to a tolerable norovirus infection risks of 0.14 pppy.

### Data analysis

Data analysis was done using STATA 12, the WHO QMRA model and @Risk 6 (Palisade Corporation, NY-USA). All *E. coli* concentrations were Log_10_ transformed for calculations of medians and inter quartile range (IQR) for irrigation water samples and means, standard deviations and 95 % confidence intervals (CI) for soil samples. Unlike irrigation water, *E. coli* concentrations in soil were normally distributed after log transformation. The Mann–Whitney and Kruskal-Wallis tests were used to test the association of risk factors with irrigation water quality. One-way anova and two sample *t*-test were used to assess the effect of risk factors on soil contamination. Only factors that were significant at 10 % in the univariable analysis were included in a multiple regression model that was used to identify risk factors for soil contamination. Statistically significant differences between exposures and outcomes in the multiple regression model were measured at 5 % significance level using likelihood ratio test. Irrigation water was also reclassified as a binary variable (≤3 Log *E. coli*/100 ml and > 3 Log *E. coli*/100 ml) representing the water quality guidelines set by the WHO [20]. The proportion of time farmers worked unprotected for each farm activity was determined as a proportion of the time they work unprotected over the total time used to undertake the activity. The observed annual contact time for each type of contact (e.g. feet-to-soil), was calculated as the product of the total daily contact time for all observed farm activities, the number of days farmers work within a month and the months they work in a year. The expected dose of *E. coli* likely to be ingested due to hand-to-mouth events was estimated from a Poisson distribution [21] and Monte Carlo simulation using the soil quality from this study and the range of soil quantity (10 - 100 mg/d) assumed to be accidentally ingested by farmers. Regarding the norovirus infection risk to farmers, only the median and 95th percentile risk per person per year were reported after the Monte Carlo simulation.

## Results

### Irrigation water and farm soil quality and risk factors

During the survey only 7 % of the 160 irrigation water samples and 9 % of the 163 soil samples were found to be free from *E. coli*. Overall, the median concentration of irrigation water was 5.6 Log *E. coli*/100 ml, while the mean concentration of soil was 2.3 Log *E. coli*/g.

The use of poultry manure, the quality of irrigation water and time since soil was last irrigated were all associated with increased levels of soil contamination in the univariable analysis (Table 2). In the multivariable analysis the effect of irrigation water and seasonality remained strongly associated with the levels of soil contamination after controlling for the use of chicken manure. There was interaction between seasonality and irrigation water quality which was significant in the multivariable analysis (*p* = 0.02, 95 % CI = -0.36, -0.04) and resulted in higher levels of soil contamination in the dry season than in the rainy season (Table 2). For irrigation water, univariable analysis showed significant differences in the *E. coli* quality among the different types of irrigation water with drain water being the most contaminated (*p* < 0.001, Table 2).

**Table 2:** *E. coli* contamination of irrigation water and farm soil

Water quality	N	Median	IQR***	P_1_
* E. coli* (Log10/100 ml)				
Dry season	80	5.37	3.61, 6.27	0.35
Rainy season	80	5.73	3.48, 6.61	
Water sources				
Drain water	36	6.61	5.93, 6.81	<0.001
Dug-out	41	3.78	3.00, 5.69	
Piped water	3	2.65	2.65, 3.30	
Proximity to garbage				
≤3 m	59	5.90	3.70, 6.72	0.02
>3 m	21	4.57	3.00, 5.79	
Farm soil parameter	N	Mean (SD*)	95 % CI**	P_2_
* E. coli*(Log10/g)				
Dry season	83	2.25 (0.93)	2.05, 2.46	0.93
Rainy season	80	2.24 (0.92)	2.04, 2.45	
With manure (both seasons)				
Yes	128	2.34 (0.89)	2.19, 2.50	0.01
No	33	1.90 (0.94)	1.57, 2.23	
Irrigated with:				
Drain water	36	2.84 (0.61)	2.63, 3.04	<0.001
Dug-out	41	1.79 (0.86)	1.52, 2.06	
Piped water	3	1.27 (0.53)	−0.06, 2.59	
When irrigated				
≤1 day	32	2.58 (0.90)	2.26, 2.91	0.01
Between 1 day – 2 days	13	2.11 (0.83)	1.61, 2.61	
>2 days	35	1.98 (0.90)	1.67, 2.29	
Multivariable analysis				
Exposure	N	Change in mean	95 % CI**	P_3_
Irrigation water	160	0.41	0.30, 0.52	<0.001
Manure	163	0.23	−0.11, 0.57	0.03
Seasonality	160	0.97	0.11, 1.85	0.05
Season #irrigation water	160	−0.20	−0.36, -0.04	<0.001

SD* standard deviation

95 % CI** 95 % confidence interval

IQR*** = Interquartile range

P_1_, p-value calculated using Mann–Whitney test or Kruskal-Wallis test for irrigation water quality. P_2_, p-value calculated using *t*-test and Anova for farm soil quality

P_3_, p-value calculated using likelihood ratio test

### Farmer observations

Of the 80 farmers all but 4 were male with an average age of 40 years (range 22 – 72). Agriculture formed the main source of income for the large majority of farmers (80 %), while over 70 % of farmers were literate. There were no toilet facilities found on the sites and 73 % of farmers reported that they practiced open defecation when working at their fields (Table 3). The majority (77 %) of farmers ate their food in their fields and mostly consumed it cold after keeping the food over a period of time.

**Table 3:** Characteristics at farm sites

Exposure	N (%)
Poultry manure use*	
Dry season (*n* = 80)	48 (60)
Rainy season (*n* = 83)	82 (99)
Last irrigated (*N* = 80)	
≤1 day	32 (40)
1 to 2 days	13 (16)
2 to 3 days	21 (26)
>3 days	14 (18)
Defecation practices of farmers	
Public toilet	20 (25)
Neighbour’s toilet	2 (2.4)
Open Defecation	58 (73)
Source of farmers drinking water	
Sachet water	58 (73)
Piped water	22 (27)
What used to wash hands before eating (*N* = 76)	
Irrigation water only	2 (2.6)
Piped water only	22 (29)
Sachet water only	8 (11)
Water and soap	44 (58)
Observed hand washing practices before eating (*N* = 21)	
Washed hands before eating	17 (81)
Washed hands with water and soap	1 (5.9)
Washed hands with only water	16 (94)
Whether drain water increases farmers income compared to piped water	
Yes	48 (60)
No	3 (4)
Cannot tell	29 (36)
Farming as main source of income	
Yes	63 (79)
No	17 (21)
Where farmers eat often when at work (*N* = 77)	
On the farm	59 (77)
At vending sites	8 (10)
At home	10 (13)
Reported working times in the field	
Average daily working hours (min – max)	7.1 (4, 13)
Average days worked per week (min – max)	6.7 (5, 7)
Average days worked per month (min – max)	28.2 (20, 30)
Average months worked per year (min – max)	11.8 (9, 12)
Average days worked per year (min – max)	336.7 (240, 360)

* p-value, < 0.001

**Fig. 1:**
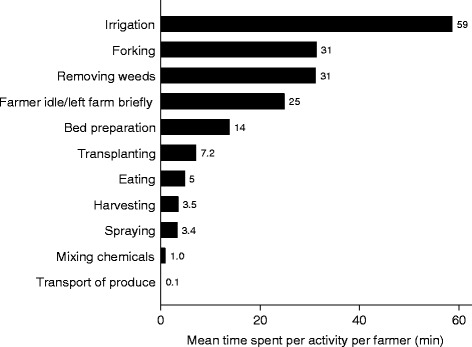
Observed time (3 hours) for undertaking farm activities

**Fig. 2:**
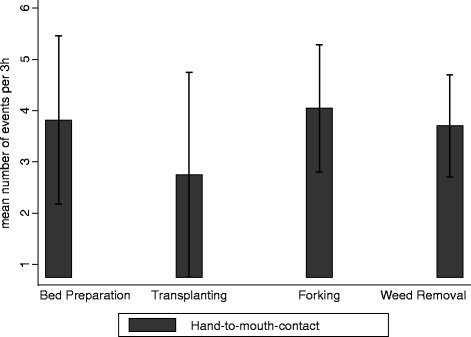
Observed farmers’ hand-to-mouth contact events per 3 hour observation period, by farm activity. * *Error bars represent 95 % CI of the mean*

A total of 10 farming activities were observed during the 3 h, though the majority (79 %) of time was spent on 5 key activities, with irrigating the most common (33 % of total time) (Fig. 1). During the observation period almost all farmers (97 %) were observed to have hand-to-soil contact and 89 % of farmers were found to work bare-foot in their field for any of the activities (Table 4). In addition over 90 % of farmers involved in irrigation had their hands and feet exposed to irrigation water. The number of hand-to-mouth contacts per farmer was highest during “forking” with an average of 4 events/3 h, but ranged from 1 to 12 events (Fig. 2). For all activities observed within the 3 h, 86 % of farmers experienced hand-to-soil contact for an average time of 100 min while 93 % worked bare-foot for 145 min (81 % of total time). In addition 63 % of farmers had both feet and hands exposed to irrigation water for at least 88 min (49 % of total time).

**Table 4:** Farmers’ annual contact time to irrigation water and farm soil per contact type and farm activity

Variable	% of farmers involved in activity at peak period % (*N* = 80)	Percentage of farmers with contact to faecal contamination, % (n)	Median (IQR) contact time, h/y
*Bed Preparation (n = 19)*	30 (24)		25 (16, 39)
Hand-to-soil		100 (19)	24 (14, 39)
Feet-to-soil		90 (17)	24 (16, 33)
Hand-to-mouth/face, nr/y*		58 (11)	85 (45, 227)
*Transplanting (n = 11)*	18 (14)		18 (13, 45)
Hand-to-soil		100 (11)	18 (13, 45)
Feet-to-soil		100.(11)	18 (13, 45)
Hand-to-mouth/face, nr/y		36 (4)	85 (57, 270)
*Soil tilling (Forking, n = 36)*	56 (45)		150 (83, 290)
Hand-to-soil		97 (35)	152 (84, 308)
Feet-to-soil		92 (33)	144 (81, 273)
Hand-to-mouth/face, nr/y		61 (22)	454 (227, 852)
*Weed Removal (n = 42)*	66 (53)		99 (47, 189)
Hand-to-soil		100 (42)	99 (47, 189)
Feet-to-soil		98 (41)	95 (47, 189)
Hand-to-mouth/face, nr/y		48 (20)	256 (128, 852)
*Irrigation (n = 55)*	86 (69)		1113 (426, 1617)
Hand-to-irrigation water		93 (51)	1278 (451, 1633)
Feet-to-soil		89 (49)	1278 (450, 1633)
Feet-to-irrigation water		91 (50)	1295 (451, 1633)
Total hand-to-soil contact**	100 (80)	86 (69)	1339 (909, 1732)
Total feet-to-soil contact^†^	100 (80)	93 (74)	2002 (1625, 2300)
Total hand-to-mouth contact events	100 (80)	53 (42)	3181 (1704, 5964)

nr/y* = number of events per year

** Total hand-to-soil contact for 5 farm activities – bed preparation, transplanting, soil tilling, weed removal and harvesting

^†^ Total feet-to-soil contact for 8 farm activities - bed preparation, transplanting, soil tilling, weed removal, irrigation, spraying, harvesting and transport of produce to roadside

### Reported exposure frequencies and risk practices

**Fig. 3:**
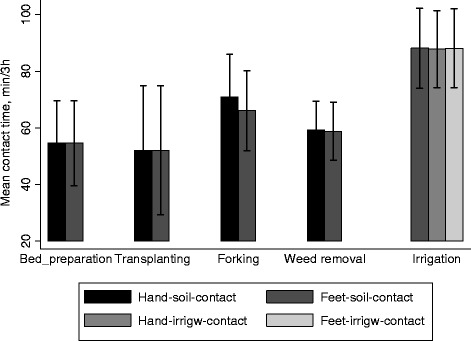
Observed farmers contact time (minutes) per 3 hour observation period, by contact type and farm activity. * *Error bars represent 95 % Cl of the mean*

Farmers reported to work an average of 7.1 h per day, 28.2 days per month and 11.8 months per year on their farm (Table 3). These then translate to an average annual time spent working in the field of 337 days, or 2,393 hours. In terms of farming activities, farmers spend a median of 720 hours and a maximum of 2,880 hours per year (120 days) irrigating, though irrigation was done on average 324 days (27 days per month) per year (Table 5). Of the five major farm activities, irrigation recorded the highest feet-to-soil contact at 88 min/3 h (Fig. 3) which translated into annual median contact of 1,278 h/y (Table 4). The observed median feet-to-soil contact for farm activities was 2,002 h/y (maximum of 2,556 h/y, or 107 d/y). “Forking” had the highest hand-to-soil contact time (53 %, 152 h/y) while no hand-to-soil contact was observed during irrigation. Every day, farmers had a median of 10 hand-to-mouth events (3,181/y).

**Table 5:** Farmers’ reported annual working time per farm activity

Farm activity	Farmers	Average frequency of activity, d/m	Median (IQR) (h/y)	Min – Max (h/y)
Bed preparation	79	1.1	24 (12, 36)	2.25, 192
Transplanting	79	1.1	27 (18, 48)	3, 240
Soil tilling (“Forking”)	80	6.9	180 (96, 219)	24, 528
Removing weeds	80	5.6	96 (48, 174)	12, 720
Irrigation	80	27.0	720 (360, 1080)	72, 2880
Manure application	79	1.3	12 (9, 24)	3, 135
Total time for 6 activities	79	NA	1062.1 (771, 1634.4)	282, 3396

*NA* not applicable

Although all farmers reported to wash their hands before eating, only one (6 %) of those observed to wash their hands before eating was found to have used soap (Table 3). The use of poultry manure was common, though significantly higher among farmers in the dry season than in the rainy season (Table 3). Only a few farmers (6 %) were seen using gloves, or masks (1 %), though a slightly higher proportion of farmers reported that they used gloves and masks while preparing chemicals for spraying crops (9 % vs. 6 %). Irrigation cessation was not reported to be practiced, though the average irrigation frequency went down to every other day (56 h) during the rainy season (Table 3).

### QMRA and risk to farmers

From the Poisson distribution, farmers could ingest a minimum of 2 *E. coli/*d and a maximum of 18 *E. coli*/d if they ingested soil of average contamination 2.3 Log *E. coli*/g (Table 6). For the maximum soil contamination of 4.1 Log *E. coli*/g, farmers could ingest between 126 *E. coli*/d and 1,259 *E. coli*/d. Farmers were likely to spend an average contact time of 103 d/y and a maximum of 132 d/y in direct contact in the fields for all activities, assuming 100 % feet contact to soil. The median norovirus infection risk for farmers exposed to soil of quality 2.3 Log *E. coli*/g and ingesting between 10 – 100 mg/day soil was estimated to be 8.5 × 10^-3^ pppy and 3.4 × 10^-3^ pppy for exposures of 337 days and 132 days respectively (Table 7). When exposed to soil of the highest contamination (4.1 Log *E. coli*/g), the median norovirus infection risk for farmers increased to 0.42 pppy; and 0.19 pppy for reported and observed exposures of 337 days and 132 days respectively (Table 7).

**Table 6:** Daily dose of *E. coli* ingested by farmers ingesting 10 – 100 mg of soil

Soil quality (Log *E. coli*/g)	Soil ingested (mg)	Dose of *E. coli* ingested	
		Mean dose	95 % Confidence interval
Average soil concentration			
10^2.3^	10	1.8	0, 5
	100	17.8	10, 27
Max. soil concentration			
10^4.1^	10	126	104, 149
	100	1,259	1189, 1327

**Table 7:** Median norovirus infection risks to farmers from the involuntary ingestion of 10 - 100 mg of wastewater-saturated soil per day for 337 and 132 days per year estimated by 10, 000 Monte Carlo (MC) simulations

Soil quality (*E. coli*/g soil)	^d^Reported exposure frequency (337 days exposure)		Observed exposure frequency (132 days exposure)	
	^a^Median norovirus risk pppy	95-percentile norovirus risk pppy	^a^Median norovirus risk pppy	95-percentile norovirus risk pppy
10^4.1b^	0.42	0.44	0.19	0.21
10^3^ – 10^4^	0.21	0.23	9.3 × 10^-2^	1.0 × 10^-1^
10^2^ – 10^3^	2.3 × 10^-2^	2.5 × 10^-2^	9.7 × 10^-3^	1.1 × 10^-2^
10^2.3c^	8.5 × 10^-3^	9.1 × 10^-3^	3.4 × 10^-3^	3.7 × 10^-3^
10^1^ – 10^2^	2.4 × 10^-3^	2.6 × 10^-3^	9.7 × 10^-4^	1.1 × 10^-3^

^a^Karavarsamis-Hamilton MC simulations. Assumptions: 0.1-1 norovirus per 10^5^ *E. coli*, no pathogen die-off, disease/infection ratio 1:1, ^b^ Maximum soil contamination, ^c^ Average soil concentration. ^d^Reported exposure frequency reflects only the days farmers report in the field but does not necessarily reflect the actual time farmers spent in the field, or were engaged in risky activities that expose them to faecal pathogens (observed exposure frequency)

## Discussion

This study found high concentrations of *E. coli* in irrigation water, though exposure to soil posed the highest risk as a result of frequent hand to soil and hand to mouth contacts especially during weeding and forking. Based on the WHO developed QMRA models, farm practices in Accra exceeded maximum recommended disease risks.

### Irrigation water and farm soil quality

The study found that irrigation water sources used for vegetable cultivation were highly contaminated, with 84 % of water samples exceeding the WHO water quality standard of 3 log *E. coli*/100 ml for unrestricted irrigation [20]. The high concentrations of *E. coli* found in irrigation water were similar to those found previously in Ghana [22, 23], though significantly lower than those found in India and Pakistan where farmers were found to use untreated wastewater [24]. Unlike in Pakistan where farmers used raw sewage from a wastewater treatment plant, wastewater used by farmers in Accra was diluted by rainwater, or other sources of storm water. Although water quality was the main factor affecting the presence and concentrations of *E. coli* in soil, the use of poultry manure further contributed to increased levels of *E. coli* in soil. The concentrations of *E. coli* found in soil were lower (2.3 Log *E. coli*/g vs. 3.0 Log faecal coliform/g) than those found previously in Ghana [22], and this could be due to differences in the microbial quality of irrigation water, or the frequency of manure application to soil between the two studies. *E. coli* is an indicator organism for faecal contamination and the high concentrations found in irrigation water and farm soil are likely to indicate the presence of a variety of pathogens. Nevertheless, only few studies have enumerated the actual concentrations of pathogens, including viruses in wastewater used for irrigation due to high cost and poor viral detection efficiencies [25].

### Faecal contamination exposure pathways

The WHO QMRA model calculates permissible disease, or infection risk for farmers using wastewater on the accidental ingestion of wastewater contaminated soil during agricultural activities. However there is little evidence to support the assumption that the key risk to farmers is through the soil route, though one study has reported that agriculturalist and archaeologists have higher soil interaction than other workers [26] and that all members of an exposed population will involuntarily ingest at least small quantities of soil adhering to the skin of fingers because of hand-to-mouth activity [27]. This study found the highest concentrations of *E. coli* in irrigation water and significantly lower concentrations in soil. Farmers however, spent a higher proportion of their time in contact with soil (>80 %) than with irrigation water (49 %). In this study farmers were observed to have direct hand to soil and hand to irrigation water contacts, though hand to mouth events were only observed during soil related activities and not during irrigation. These findings therefore support the WHO QMRA model approach that is based on the accidental ingestion of soil.

The use of watering cans could possibly prevent, or limit farmers’ direct hand to mouth contact of irrigation water since farmers rarely put the watering cans down during irrigation. On the other hand, farmers could ingest some wastewater when engaged in other forms of irrigation application such as spray or sprinkler irrigation. Farmers’ prolonged exposure to wastewater could be more significant when investigating pathogens, or chemical risks that occur via dermal contact rather than through ingestion, especially when exposure to wastewater has been identified as a major risk factor for skin disease in Vietnam [28].

In terms of soil ingestion, the study found that farmers were likely to ingest between 2 *E. coli* and 1200 *E. coli/d*. This study did not isolate specific strains of *E. coli* or pathogens and was therefore unable to determine whether the exposure rates observed are likely to result in an adverse health effect since big differences exist in the infective dose for different *E. coli* strains. Again, the presence of *E. coli* only indicates the presence of faecal pollution and does not necessarily guarantee the presence of pathogens that can cause diseases to humans.

### High risk farming activities

All major farm activities were found to expose farmers to faecal contamination, though irrigation, forking and weeding were regarded as the key risk activities. The large amount of time spent by farmers on irrigation comes as a result of the manual method of irrigation application and the long distances farmers walk to access irrigation water. In this study, farmers spent about 80 % of their total working time accessing irrigation water or irrigating, and was higher than previous estimates (40 to 70 %) in Accra and Kumasi [12, 29]. Only 7 % of farmers in this study were seen to wear boots, and often only for short periods while irrigating, as was shown by studies in Kenya, Pakistan, and Côte d’Ivoire where between 5 and 19 % of farmers reported to wear boots, often citing discomfort, heat and the muddy fields as reasons why they did not wear footwear [8, 9, 30]. In India and Pakistan hookworm infection was found to be the main infection associated with the use of wastewater by farmers and the lack of use of footwear was cited as one of the main risk factors [8, 31]. In Ghana, stool surveys among wastewater farmers have not been conducted and as a result it is unknown whether irrigation practices and the lack of footwear affect hookworm prevalence.

“Forking” and weeding were found to be the major farming activities associated with farmers’ risk of accidental ingestion of soil. This was due to the high frequency of hand-to-mouth events associated with these two activities, which are often undertaken simultaneously using hand-held weeding knives and the bare hands. Farmers’ hands become contaminated as they remove weeds, stones and other waste materials, and this coupled with frequent wiping of sweat from the face due to the heat and the strenuous activities and the consumption of food make these high-risk activities. The risk of faecal pathogen transmission due to the consumption of food with contaminated hand is, however, not limited to forking and weeding but is common to other farm activities including irrigation. In addition, there is the likelihood of the soil being attached to the farmers’ hands and feet for some time after the soil related activities, and this could also present some health risks to the farmers and those that they come into contact with. The use of chicken manure was reported by between 60 and 99 % of farmers in this study and in other studies by between 70 and 98 % of farmers in Ghana [32]. The high concentrations of *E. coli* in manure [22, 32] and the fact that the manure is often applied without the use of protective clothing makes this another key health risk that is not included in the QMRA assessment and would not only apply to wastewater farmers but also to regular farmers.

### Health risks and the WHO guidelines and policy implications

This study found that the use of wastewater in Accra (and potentially other places with similar settings) exceeded the WHO permissible norovirus infection risk (1.4 × 10^-3^ pppy) corresponding to a DALY burden of 10^-6^ pppy. Similar findings were reported by Mara and Sleigh [33] and Mara et al., [6], where wastewater farmers’ norovirus infection risk exceeded guideline thresholds by at least one order of magnitude if they ingested 1–10 mg, or 10–100 mg of wastewater saturated soil for 100 and 300 days respectively. An earlier study in Accra, also found farmers’ risk of rotavirus infection (7.6 × 10^-2^) to exceed guideline value for rotavirus diarrhoea in developing countries (7.7 × 10^-4^) by 2 orders of magnitude, after ingesting 10–100 mg of soil for 150 days [34]. The fact that some studies assume a fully-saturated wastewater soil and substitute wastewater quality for soil quality could however, lead to bias results, as other contaminants such as wild animals and birds have been identified to contribute to soil quality [35].

In the current study, farmers’ risk was found to diminish by at least 50 % if an actual observed exposure time to water and soil (132 days), or contact in the field was used. Although the use of self-reported time could lead to overestimation of farmers’ risk; this influence might be more significant when assessing risk transmitted via dermal contact and not through oral ingestion. A better approach to estimate farmers’ risk due to soil ingestion would be the use of actual hand-to-mouth contact (manuscript in preparation) since these events depend more on the type of farm activity performed and not necessarily on how much time farmers spend in the field.

Currently the WHO QMRA model does not consider farmers’ risk via dermal contacts (e.g. hookworm infection). This exposure route could be particularly important in the QMRA model as almost all farmers in this study were found to be working bare-feet for most of the time, though this is less relevant for oral ingestion. For this type of transmission route the use of the direct observed contact time would be more appropriate since the self-reported time does not necessarily reflect the actual time farmers spent in the field or were engaged in risky activities that expose them to faecal pathogens. Further studies in the form of repeated observations or longer observations over the course of the year would be required to confirm farmers actual contact time to faecal pathogens and to better understand their risk behaviours and practices. This is particularly necessary as direct exposure frequency to faecal pathogens in this study was only based on a single 3 h observation per farmer, and also excluded contact to faecal matter during manure application.

The maximum permissible additional disease risk has been under discussion with some arguing that it is too strict for wastewater use in agriculture [19]. This study showed that farmers’ occupational risk was within acceptable limits for a DALY burden of 10^-4^ pppy but not for the current guideline of DALY burden of 10^-6^ pppy. Only the risk corresponding to the highest soil contamination for an exposure of 337 days exceeded this tolerable risk. A DALY burden of 10^-4^ pppy also sets the tolerable number of infections associated with wastewater exposure, though it does not by itself determine the likelihood of pathogen infections.. One of the reasons for the use of a relaxed DALY of either 10^-5^ pppy or 10^-4^ pppy was that the resulting norovirus/rotavirus disease risk would still be lower than the actual global incidence of diarrhoeal disease of 0.1 – 1 pppy in both developed and low and middle-income countries [36]. In addition, it would result in a reduction in the cost required for wastewater treatment; and hence the extra money saved could be used for other risk reduction interventions.

Although high, the estimated risk from this study should be interpreted with caution. First, the risk arising from the mean soil quality was found to safe, though soil samples (51 %) with quality just above the mean (2.4 Log *E. coli*/g) resulted in a risk higher than the guidelines limit. Even with this quality, farmers’ risk would still be within the acceptable limits, or would be marginally safe if exposure (ingestion) to contaminated soil was at most 300 days (9.0 × 10^-3^ pppy).

There were limitations of the model that was used to estimate the risk. The model used published ratios between *E. coli* and norovirus and not necessarily the actual concentrations of norovirus. The use of these ratios often assume a linear relationship between the indicator organism and the pathogen and also ignore other factors such as seasonality, transport characteristics of microbes and other environmental factors which could influence this correlation. There is also inadequate evidence to support the widely used ratio of 1:10^5^*E. coli*/faecal coliform to virus relationship, which was based on a study in northeast Brazil [37]. A recent study in Ghana found an average of one norovirus GII to 10^3.2^*E. coli* or 10^4.8^ thermotolerant coliforms, from its quantifiable irrigation water samples, which suggest that the NV-GII to *E. coli* ratio is much lower than the widely used ratio of 1:10^5^ [23]. In the current study, the ratio of means between norovirus and *E. coli* was estimated as 1:10^1.7^ (1.9 × 10^1^ genome copies/mL vs. 8.9 × 10^2^*E. coli*/mL, *N* = 67) from irrigation water samples analysed for both *E. coli* and norovirus, which is also much lower than the common ratios used in recent publications. The above observation also means that the estimated number of NV-GII in the current study or the previous study in Ghana would be higher than if it were estimated from the higher NV-GII to *E. coli* ratio of 1:10^5^.

The other limitation is that the study did not assess for helminths and protozoans and hence the model could underestimate farmers’ risk, though the risk associated with viruses is generally considered high enough to adequately protect farmers from bacterial and protozoan infections. The use of soil quality in the risk model instead of water quality as used in some other studies is, however, considered as the “closest” and a better estimate of farmers’ risk due to faecal-oral ingestion. A third limitation is that the study was unable to estimate the actual mass or quantity of soil likely to be ingested by farmers and therefore still relied on the range normally for QMRA estimations from the literature. A further refinement of QMRA input data would be to estimate the total mass of soil ingested but also classify the exposure mass of soil based on the different types of farming activities such as transplanting and weeding.

An updated version of the WHO QMRA model should incorporate actual hand-to-mouth events in the model since these models often deal with ingestion of contaminated products such as soil, irrigation water and produce. In terms of pathogen reductions, farmers’ risk of 0.42 pppy means that reducing the contamination of irrigation water by two to three log units per 100 ml irrigation water or 100 g soil (assuming a fully saturated soil) will keep farmers occupational health risk within acceptable levels for a DALY burden of 10^-6^ pppy. A significant part of soil contamination was attributed to the use of chicken manure, and hence adequate treatment of the manure before application is also recommended to reduce farmers’ risk, in particular those caused by zoonotic pathogens, such as Campylobacter; further, manure safety management should form part of the WHO guidelines.

Although these reductions in microbial contamination can be achieved by simple wastewater treatment such as the use of the three-tank or three-pond system which is operated as a sequential batch-fed process [33]; in the short term, wastewater treatment seems an unlikely intervention as farmers are unable to invest in wastewater treatment due to insecure land tenure system, and the high costs. Farmers are also unlikely to allow their irrigation water to settle for 6 days to reduce thermotolerant coliforms levels as recommended by Keraita *et al* [38] due to the long waiting periods and the fact that farmers seem more concerned about keeping their produce fresh for higher yields and profits. Instead, local authorities and other stakeholders should collaborate with farmers by providing credit or loan schemes and also increase land security to farmers who adhere to agreed and prescribed safe practices. This in turn could motivate farmers to invest more in on-farm risk reduction measures such as on-farm sedimentation ponds, and also adopt other good agriculture practices as well as personal and environmental hygienic practices that could reduce both occupational and consumer risk.

## Conclusion

This study found exposure to soil as the critical pathway of pathogen risk in wastewater farmers as a result of hand-to-mouth events, and hence the findings validate the WHO QMRA approach which bases farm risks on the accidental ingestion of soil. Farm practices were also found to exceed the WHO health based target of ≤ 10^-6^ DALY burden pppy; though the limitations of the model make the results inconclusive to provide sufficient evidence on the actual risk to wastewater farmers. The study recommends the incorporation of hand-to-mouth soil events in QMRA models and the use of actual pathogen concentrations in soil and in irrigation water to estimate farmers’ risk. Apart from faecal-oral transmission, the study also recommends models for other transmission pathways such as dermal contacts especially in settings with high prevalence of hookworm. Lastly, for any of the disease or pathogen transmission pathways, and based on the study findings, the study recommends the use of a much lower exposure frequency for contact with soil (~150 d/y) or wastewater (120 d/y) by agricultural workers and also a relaxed DALY burden of 10^-4^ pppy especially for Ghana and other low and middle income countries considering the many other transmission routes, of which transmission via occupational exposure to wastewater is just one of them.

## Abbreviations

CFU, Colony Forming Unit; DALY, Disability Life Adjusted Years; ha, hectare; NV-GII, Norovirus genogroup II; PBS, Phosphate buffered saline; pppd, per person per day; pppy, per person per year; QMRA, Quantitative Microbial Risk Assessment; WHO, World Health Organisation.
